# Altered Mitochondria Morphology and Cell Metabolism in Apaf1-Deficient Cells

**DOI:** 10.1371/journal.pone.0084666

**Published:** 2014-01-09

**Authors:** Mónica Sancho, Anna Gortat, Andrés E. Herrera, Vicente Andreu-Fernández, Elisabetta Ferraro, Francesco Cecconi, Mar Orzáez, Enrique Pérez-Payá

**Affiliations:** 1 Laboratory of Peptide and Protein Chemistry, Centro de Investigación Príncipe Felipe, Valencia, Spain; 2 Laboratory of Skeletal Muscle Development and Metabolism, IRCCS San Raffaele Pisana Institute, Rome, Italy; 3 Laboratory of Molecular Neuroembryology, IRCCS Fondazione Santa Lucia, Rome, Italy; 4 Dulbecco Telethon Institute, University of Rome ‘Tor Vergata’, Rome, Italy; 5 Instituto de Biomedicina de Valencia, IBV-CSIC, Valencia, Spain; Roswell Park Cancer Institute, United States of America

## Abstract

**Background:**

Apaf1 (apoptotic protease activating factor 1) is the central component of the apoptosome, a multiprotein complex that activates procaspase-9 after cytochrome *c* release from the mitochondria in the intrinsic pathway of apoptosis. Other cellular roles, including a pro-survival role, have also been described for Apaf1, while the relative contribution of each function to cell death, but also to cell homeostatic conditions, remain to be clarified.

**Methodology and Principal Findings:**

Here we examined the response to apoptosis induction of available embryonic fibroblasts from Apaf1 knockout mice (MEFS KO Apaf1). In the absence of Apaf1, cells showed mitochondria with an altered morphology that affects cytochrome *c* release and basal metabolic status.

**Conclusions:**

We analysed mitochondrial features and cell death response to etoposide and ABT-737 in two different Apaf1-deficient MEFS, which differ in the immortalisation protocol. Unexpectedly, MEFS KO Apaf1 immortalised with the SV40 antigen (SV40IM-MEFS Apaf1) and those which spontaneously immortalised (SIM-MEFS Apaf1) respond differently to apoptotic stimuli, but both presented relevant differences at the mitochondria when compared to MEFS WT, indicating a role for Apaf1 at the mitochondria.

## Introduction

Apoptosis is an essential process of programmed cell death for normal development, cell homeostasis, and also as a defence mechanism to eliminate harmful cells, such as tumour cells or cells infected by viruses. It is characterised by specific morphological changes, such as shrinkage of cell and chromatin condensation. Apoptosis can be triggered by extrinsic (death receptor-mediated [1]) or intrinsic (mitochondrial) pathways. The intrinsic pathway can be initiated by many stresses [2], and both pathways can provoke mitochondrial outer membrane permeabilisation (MOMP) mediated by proteins of the Bcl-2 family (Bcl-2s). Cytochrome *c* (Cyt *c*) is then released into the cytosol and induces the formation of the apoptosome complex. The apoptosome is a holoenzyme complex formed by Cyt *c*-activated Apaf1, dATP and procaspase-9 [3], [4]. Apoptosome-bound procaspase-9 is activated, and subsequently proteolyses and activates downstream effector caspases, leading to the progression of cell death. Apaf1 is a 135 kDa protein that is known for its apoptotic role. However, recent studies have suggested additional non-apoptotic functions for Apaf1, including a pro-survival role [5]–[10]. How these, in principle, opposite functions of the protein operate in cells remains a controversial matter, and there are still important questions to answer about the Apaf1 biological function, not only when cells die, but also under homeostatic conditions.

Although the cells deriving from Apaf1 knockout (KO) animals are expected to be resistant to the majority of apoptotic insults, different laboratories have presented evidence that some, but not all, immortalised Apaf1-deficient cell types can switch between apoptotic and necrotic cell death [11]–[14]. These differences might be related to an influence of the immortalisation process on the availability of cell death signalling components [15], [16] or to a more complex role of Apaf1 in the cell than expected. In addition, the relationship between the extent of Cyt *c* release from the mitochondria and the completeness of downstream apoptotic signalling is still controversial. Studies at the single cell level have provided clear evidence for a single-step release mechanism of Cyt *c* and of other mitochondrial proteins, such as Smac/DIABLO, even in Apaf1-deficient cells [17], [18]. Moreover in cells of different origins lacking Apaf1, it has been reported that Cyt *c*-release is either inhibited [12] or, in contrast, it increases [9]. Furthermore, the pharmacological inhibition of Apaf1 has been reported to induce a reduction in the total amount of Cyt *c* released from a cell population [7].

Here we report a profound characterisation of available embryonic fibroblasts from Apaf1 KO mouse (MEFS KO Apaf1). We found that distinct MEFS KO Apaf1 cells behave differently in response to apoptotic insults. We analysed the apoptotic response to such insults, as well as the mitochondrial and metabolic status in MEFS KO Apaf1, which were spontaneously immortalised (SIM) or immortalised by the transfection of the SV40 antigen (SV40IM). In the absence of Apaf1, cells present mitochondria with an altered morphology which affects Cyt *c* release and basal metabolic status.

## Materials and Methods

### Cell culture, treatments and chemicals

All the cell lines were grown in Dulbecco's Modified Eagle's Medium (DMEM) supplemented with 10% foetal bovine serum (FBS). Cultures were maintained at 37°C in a 5% CO_2_ atmosphere. Cell media and FBS were purchased from GIBCO BRL Life Technologies. When indicated, cells were treated with 5 µM of etoposide (E), acquired from Sigma Aldrich. When required, 100 µM necrostatin (Nec; Enzo Life Sciences), 10 µM SVT016426 (SVT) or 5 µM Z-Val-Ala-Asp(OMe)-fluoromethylketone (zVAD; Tocris) were administered 1 h prior to treatment addition, and cells were maintained in culture for 24 h. MEFS cell lines were previously established in the referenced publications [4], [9]. For the MEFS cells established by spontaneous immortalisation (SIM), two clones of each cell line (WT and KO Apaf1) were tested. No intrinsic variability was observed between them. Lipofectamine™ 2000 (Invitrogen) was used according to the manufacturer's instructions to transfect HeLa cells with a control random siRNA (Rsi) and Apaf1 siRNA (Asi), obtained from Cell Signaling.

### Caspase activity determination

Cell extracts were prepared from 2.0×10^5^ cells seeded in 6-well plates. After 24 h, cells were treated as indicated above, and were scraped and washed with PBS. Pellets were lysed in extraction buffer (50 mM PIPES, 50 mM KCl, 5 mM EDTA, 2 mM MgCl_2_, 2 mM DTT, supplemented with protease inhibitors). Having frozen and thawed three times, cell lysates were centrifuged at 14,000 rpm for 5 min and supernatants were collected. Quantification of the total protein concentration was performed by the BCA protein assay (Thermo Scientific). Total protein (50 µg) was mixed with 200 µL of caspase assay buffer (PBS, 10% glycerol, 0.1 mM EDTA, 2 mM DTT) containing 20 µM Ac-DEVD-afc (Enzo Life Sciences) of the caspase-3 substrate. Caspase activity was continuously monitored following the release of fluorescent afc at 37°C with a Wallac 1420 Workstation (λ_exc_ = 400 nm; λ_em_ = 508 nm). Caspase-3-like activity was expressed as the increase of relative fluorescence units per min (A.U.).

### Trypan blue exclusion assay

Cells were seeded in 6-well plates at a cellular density of 2.0×10^5^ cells/well. After 24 h, cells were treated as described before to be then detached, and 0.05% trypan blue dye was added in solution. Live cells possess intact cell membranes that exclude the dye, whereas dead cells do not. Unstained (viable) and stained (non-viable) cells were counted separately in a haemacytometer and the total number of viable cells in the population was calculated.

### Nuclear staining

The cells cultured on coverslips were stained with 300 nM 4′-6-diamidino-2-phenylindole (DAPI) solution. The morphology of cells' nuclei was observed under a fluorescence microscope (Leica Vertical DM6000) at the 350 nm excitation wavelength. Nuclei are considered to have the normal phenotype when they glow brightly and homogenously. Apoptotic nuclei can be identified by either a condensed chromatin gathering at the periphery of the nuclear membrane or a total fragmented morphology of nuclear bodies.

### Immunoblotting

Whole cell extracts were obtained by lysing cells in a buffer containing 25 mM Tris-HCl pH7.4, 1 mM EDTA, 1 mM EGTA, 1% SDS, plus protease and phosphatase inhibitors. Protein concentration was determined by the BCA protein assay. Cell lysates were resolved by SDS-PAGE, transferred to nitrocellulose membranes, blocked with 5% non-fat milk, washed with 0.1% Tween/PBS and incubated overnight with a specific primary antibody. Membranes were washed and probed with the appropriate secondary antibody conjugated with horseradish peroxidase for enhanced chemiluminescence detection (Amersham Pharmacia Biotech). The antibody against Apaf1(#611365) was acquired from BD Biosciences, Bcl-X_L_ (#2764) and Bcl-2(#2870) came from Cell Signaling, and α-tubulin antibody (#T8203) was purchased from Sigma-Aldrich.

### DIOC staining

Cells were incubated with 50 nM 3,3′-Diethyloxacarbocyanine iodide (DiOC_2_(3)) at 37°C, 5% CO_2_, for 15 minutes in 500 µL of PBS. Then, samples were analysed in a Cytomics FC 500 (Beckman Coulter) flow cytometer with 488 nm excitation using the appropriate emission filters for Alexa Fluor ® 488 dye.

### Cytochrome *c* release assay

MEFS cells were grown in 6-well plates under the same conditions described above. After 24 h of etoposide treatment, mitochondrial Cyt *c* was followed using the InnocyteTM Flow Cytometric Cytochrome *c* Release kit (Calbiochem). Cells were analysed in a Cytomics FC 500 (Beckman Coulter) flow cytometer.

### Immunofluorescence

Cells were fixed with 4% paraformaldehyde, permeabilised with 0.1% Triton X-100, and blocked in 2% gelatin in PBS. Then they were labelled with a primary antibody against Cyt *c* (SC13561; Santa Cruz), followed by anti-mouse IgG conjugated with FITC (Jackson ImmunoResearch). Images were obtained under a Leica DM 6000 microscope (Leica DC500 camera) with a 20× objective. Two hundred cells were counted and classified according to the localisation of Cyt *c* in the mitochondria (tubular morphology) or cytosol (diffuse pattern).

### Transmission electron microscopy

MEFS cells were seeded at 3×10^4^ cells per chamber on Lab-Tek chamber slides of 4 wells (Nalge Nunc International). Then, cells were fixed for 1 h in 3.5% glutaraldehyde at 37°C and postfixed for 1 h in 2% OsO4 at room temperature. Cellular staining was performed at 4°C for 2 h in 2% uranyl acetate in the dark. Finally, cells were rinsed in sodium phosphate buffer (0.1 M, pH 7.2), dehydrated in ethanol and infiltrated overnight in Araldite (Durcupan, Fluka, Buchs SG, Switzerland). Following polymerisation, embedded cultures were detached from the chamber slide and glued to Araldite blocks. Serial semi-thin (1.5 µm) sections were cut with an Ultracut UC-6 (Leica, Leica, Heidelberg, Germany), mounted onto slides and stained with 1% toluidine blue. The selected semi-thin sections were glued (Super Glue, Loctite) to araldite blocks and detached from the glass slide by repeated freezing (in liquid nitrogen) and thawing. Ultrathin (0.07 µm) sections were prepared with the Ultracut UC-6 and stained with lead citrate. Finally, photomicrographs were obtained under a transmission electron microscope (FEI Tecnai Spirit G2) using a digital camera (Morada, Soft Imaging System, Olympus). Then, mitochondrial width was quantified by using the Image J Java-based image processing software (NIH).

### ATP, lactate and pyruvate measurements

HeLa cells were seeded at a density of 5×10^4^ cells/mL. An ATP measurement was taken in duplicate by employing the Luminescence ATP Detection Assay System (Perkin Elmer) according to the manufacturer's instructions. The KO MEFs results were normalised to their respective WT controls. Lactate was measured with the Lactate Assay Kit II (BioVision) following the manufacturer's instructions. Pyruvate was measured using the Pyruvate Assay Kit (BioVision) according to the manufacturer's instructions. The KO MEFs results were normalised to their respective WT controls.

## Results and Discussion

**Figure 1:**
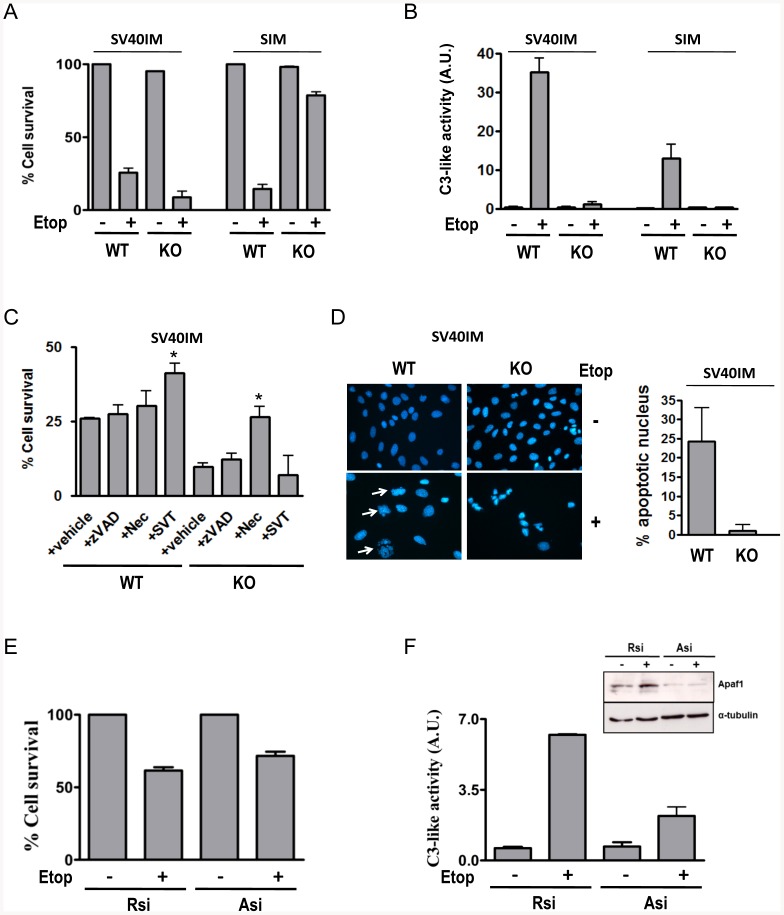
Caspase-independent cell death in SV40IM Apaf1 KO cells. (A) Cell survival was measured by the trypan blue exclusion assay in SV40IM and SIM MEFS, WT and Apaf1-depleted cells in the presence of etoposide (5 µM) for 24 h. (B) Caspase-3-like activity was measured under the same etoposide treatment conditions (+; 5 µM) described above. In all cases, bars represent the mean of three experiments ± s.d. (C) Cell survival was measured in the SV40IM cell lines treated with etoposide (5 µM) in the presence or the absence of z-VAD (5 µM), necrostatin (Nec; 100 µM) or SVT016426 (10 µM). (mean ± s.d, n = 3, *p≤0.05). (D) DAPI staining to analyse the apoptotic features between WT and Apaf1 KO cells in the presence of etoposide (5 µM). In all, 500 nuclei were counted and classified according to apoptotic nuclear bodies (white arrows). Quantification is shown in the right panel. (E) Cell survival was measured by the trypan blue exclusion assay in the HeLa cells transfected with random siRNA (Rsi) or Apaf1 siRNA (Asi) for 24 h and treated with etoposide (+; 5 µM) for another 24-hour period. (F) Caspase-3-like activity was measured under the same conditions described above. Bars represent the mean of three experiments ± s.d. The immunoblotting of the Apaf1 silencer is shown in the right panel.

The embryonic fibroblast cell lines from wild-type (MEFS WT) and from Apaf1 KO mouse (MEFS KO Apaf1) [4] were previously established by spontaneous immortalisation (SIM) or by infection with SV40 antigen T (SV40IM) [9]. The cell lines were analysed to study the relevance of Apaf1 in the homeostasis of the cell and their behaviour upon apoptotic stimuli. As an apoptotic insult, we used etoposide, a DNA damage-inducing drug that is well-characterised as an intrinsic apoptosis inducer [19]. SV40IM-MEFS KO Apaf1 cells were more sensitive to etoposide than SV40IM-MEFS WT as manifested by lower counts of cell survival determined by trypan blue exclusion (Fig. 1A). In contrast, and as initially expected [4], SIM-MEFS KO Apaf1 cells were resistant to etoposide (Fig. 1A), while SIM-MEFS WT cells showed a similar percentage of death to SV40IM-MEFS WT cells. SIM-MEFS WT and SV40IM-MEFS WT death concurred with caspase activation, while etoposide-induced death in SV40IM-MEFS KO Apaf1 proceeded without caspase activation (Fig. 1B). In etoposide-induced SV40IM-MEFS WT, caspase-3 activity diminished in the presence of the irreversible caspase inhibitor Z-Val-Ala-Asp(OMe)-fluoromethylketone (zVAD) and in the presence of the Apaf1 inhibitor SVT016426 (SVT) (not shown), while cell viability increased only in the presence of SVT016426 (Fig. 1C). Neither RIPK1 inhibitor necrostatin (Nec) nor zVAD had any effect on cell viability in these cells. In contrast in SV40IM-MEFS KO Apaf1 cells, neither zVAD nor SVT016426 influenced cell death, while Nec increased cell survival, suggesting that these cells can engage alternative death pathways as necroptosis in the absence of Apaf1. In fact nuclear morphology studies by DAPI staining showed that when cells were treated with etoposide, SV40IM-MEFS KO Apaf1 cells did not present apoptotic nuclear bodies as SV40IM-MEFS WT cells did (white arrows; Fig. 1D). Instead dead KO Apaf-1 cells showed smaller and brighter nuclei (Fig. 1D). In order to clarify which of the two cell line systems was physiologically more relevant, Apaf1 was depleted transiently by siRNA in HeLa cells (due to the difficulty of transfecting MEFS cells) and was treated with etoposide. When the HeLa cells transfected with a control random siRNA were treated with etoposide, caspase-3-like activity (Figure 1E) was observed and cell death came close to 60% (figure 1F). However in the Apaf1 siRNA-based knockdown cells, caspase-3-like activity diminished with a slight decrease in cell death of the cell population (Figure 1E and F), which might be explained by the fact that, in the absence of Apaf1, etoposide induced caspase-independent cell death in these cells, as described previously elsewhere ([11], [20] These results indicate that the SV40 immortalised and the siRNA Apaf-1 transient depleted cells behave similarly. Thus the SV40 immortalisation procedure better reflects the punctual elimination of Apaf-1 from the cell than the SIM immortalisation procedure, which probably reproduces the adaptation and selection process produced by the permanent absence of Apaf-1 in the cell. From the pharmacological point of view, both models could probably be relevant depending on the chronic or acute nature of the treatment with Apaf-1 inhibitors required for disease resolution.

**Figure 2:**
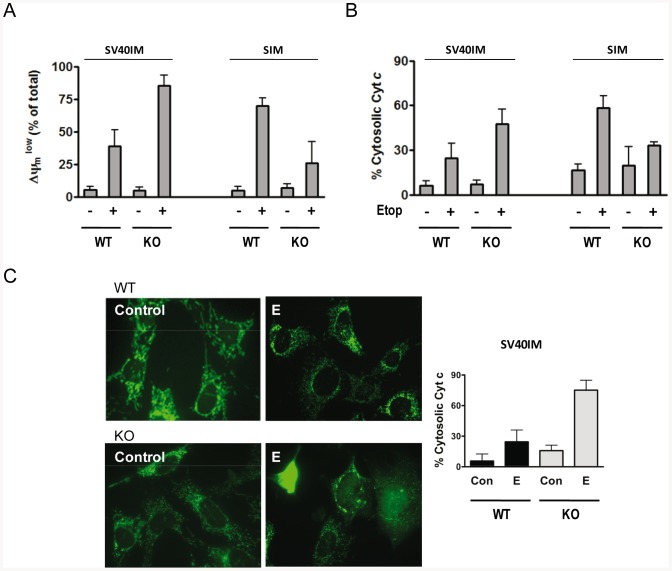
SV40IM Apaf1 KO cells showed faster Cyt *c* release. (A) Membrane potential study through DIOC_2_(3) staining in all the cell types upon etoposide treatment (+; 5 µM). (B) Percentage of cells with mitochondrial Cyt *c* measured by the cytometry analysis after incubation with etoposide (+; 5 µM) (mean ± s.d., n = 3). (C) Immunofluorescence against Cyt *c* in SV40IM WT and Apaf1 KO MEFS in the absence (Control) or presence of etoposide (E, 5 µM). In all, 200 cells were counted and classified according to mitochondrial or cytosolic Cyt *c* staining (white arrows). Quantification is shown on the right as the percentage of cells with cytosolic Cyt *c* staining (mean ± s.d., n = 3).

Next we evaluated the mitochondrial membrane potential (ΔΨm). It is well-accepted that DNA-damaging agents, such as etoposide, induce signalling processes that convey into the mitochondria by inducing Cyt *c* release and a subsequent decrease in ΔΨm [17]. With etoposide-treated SV40IM-MEFS KO Apaf1, the population of cells with low ΔΨm increased when compared to the SV40IM-MEFS WT cells (Fig. 2A). As expected from the previous cell death susceptibility results (Fig. 1A), the resistant SIM-MEFS KO Apaf1 cells showed less cells with low ΔΨm than SIM-MEFS WT (Fig. 2A). Then we examined the release of Cyt *c* from the mitochondria after the etoposide treatment in both cell line types by flow cytometry. SV40IM-MEFS KO Apaf1 released more Cyt *c* to the cytosol than the equivalent SV40IM-MEFS WT. However, SIM-MEFS KO Apaf1 released less Cyt *c* than the control SIM-MEFS WT (Fig. 2B). These results correlate well with previous death sensitivity findings (Fig. 1A, B and C) and mitochondrial membrane potential (Fig. 2A). The immunocytochemistry experiments in the SV40IM-MEFS cell lines confirmed that Cyt *c* was located mainly in the mitochondria in the absence of a cell death inducer (Fig. 2C). Nevertheless, etoposide induced Cyt *c* release from the mitochondria to the cytosol in about 30% of SV40IM-MEFS WT cells and in around 70% of SV40IM-MEFS KO Apaf1 (Fig. 2C). All these results suggest that upon etoposide induction, Cyt *c* release is enhanced in the SV40IM-MEFS KO Apaf1 cells, which is in agreement with early kinetic observations [9]. Yet due to the absence of Apaf1, the death signalling wave probably proceeds through a necroptosis-like pathway. Thus, the immortalisation process may affect the genetic background and might be responsible for the differences between both MEFS KO Apaf1 cellular models. ABT-737 treatment induced activation of caspase-3 and death in SV40IM-MEFS WT. However in SV40IM-MEFS KO Apaf-1 and SIM-MEFS, both parameters remain unaffected (Fig. S1). These results suggest that ABT737-triggering signalling is not fully perceived by the cell, while DNA damaging agents may activate alternative cell death pathways when the intrinsic pathway of apoptosis is blocked. These results indicate that the differences between both KO cell lines probably lie at the mitochondrial level and not necessarily in the nuclear signalling events responsible for apoptosis induction.

**Figure 3:**
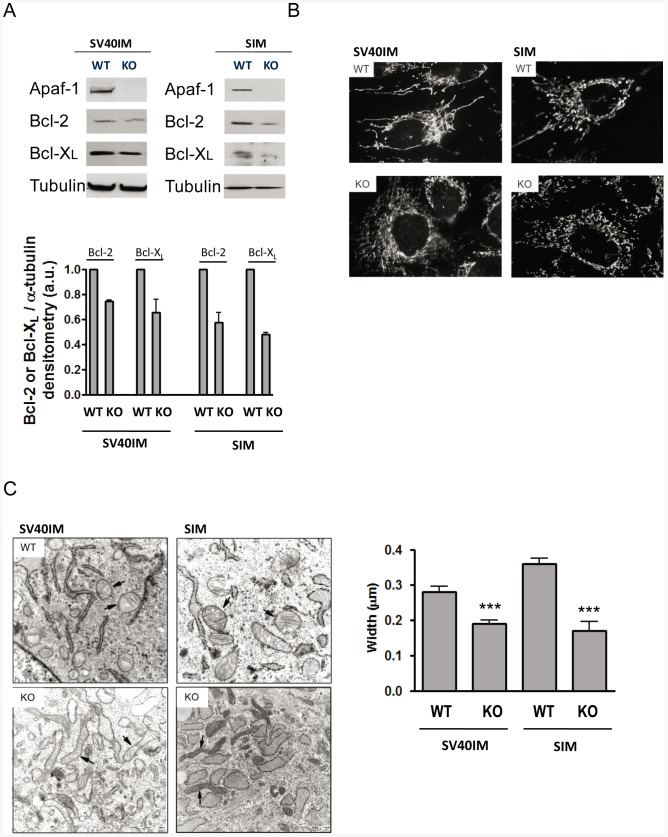
Mitochondrial network in Apaf1-deficient cells is affected. (A) Bcl-2 and Bcl-X_L_ protein levels in the SV40IM and SIM cell lines by immunoblotting. α-tubulin was also assayed as a loading control. Densitometric analyses were performed using the Image J software, normalised for α-tubulin and reported as arbitrary units (a.u). Values are means ± s.d. of three independent immunoblots. (B) The mitochondrial network in the untreated SV40IM and SIM MEF cell lines was visualised using an anti-Cyt *c* antibody. (C) Transmission electron photomicrographs of the mitochondria in MEFs. Ultrathin (0.07 µm) sections of the MEF cell lines were prepared as described in sMaterials and Method. Black arrows indicate the representative mitochondria of each sample. Scale bars indicate 1 µm. The Image J Java-based image processing software was used to quantify the width of approximately 70 different mitochondria from each cell type using the images obtained by TEM. Two-tailed Student's t-tests were performed (***p≤0.001). SV40IM-MEFS WT Apaf1 is of 0.2939±0.01501 µm and SV40IM-MEFS KO Apaf1 is of 0.1980±0.009215 µm. SIM-MEFS WT Apaf1 is of 0.3539±0.01625 µm and SIM-MEFS KO Apaf1 is of 0.1697±0.02228 µm.

**Figure 4:**
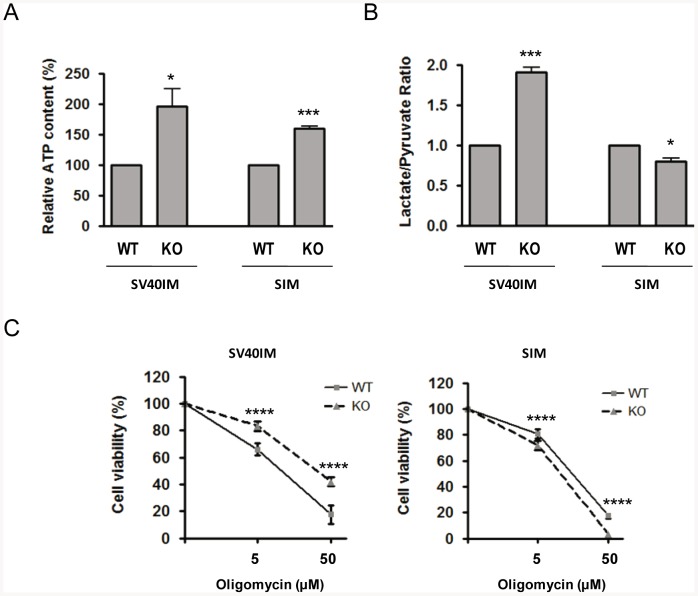
Metabolic status in Apaf1-deficient cells. (A) Absolute level of intracellular ATP in Apaf1-deficient MEFs is greater than in WT cells. ATP content was measured using the commercially available Luminescence ATP Detection Assay System (Perkin Elmer) following the manufacturer's instructions. The results obtained from each Apaf1-deficient cell line were normalised to their respective WT controls and were submitted to the two-tailed Student's t-test (*p≤0.05; ***p≤0.001).). The ATP in SV40IM-MEFS WT Apaf1 is of 100.0±0.0 and of 196.6±28.42 in SV40IM-MEFS KO Apaf1. The ATP in SIM-MEFS WT Apaf1 is of 100.0±0.0 and of 161.8±3.740 in SIM-MEFS KO Apaf1. (B) Lack of Apaf1 in MEFs affects its lactate to pyruvate (L/P) ratio. Lactate and pyruvate contents were measured using the commercially available Lactate Assay Kit II (BioVision) and the Pyruvate Assay Kit (BioVision) following the manufacturer's instructions. The results obtained from each Apaf1-deficient cell line were normalised to their respective WT controls and submitted to the two-tailed Student's t-test (*p≤0.05; ***p≤0.001). The L/P ratio in SV40IM-MEFS KO Apaf1 represents 188.9% of SV40IM-MEFS WT Apaf1 and SIM-MEFS KO Apaf1 represent 76.39% of SIM-MEFS WT Apaf1. (C) Apaf1-deficient MEFs were able to obtain ATP from both OxPhos and glycolysis. Cells from each cell line were treated with 0, 5 or 50 µM oligomycin for 24 h to block mitochondrial ATP synthase and were submitted to the MTT cell proliferation assay. The obtained results were normalised in relation to the 0 µM oligomycin control for each cell line and were submitted to the two-tailed Student's t- test (****p≤0.0001). The cell viability of SV40IM-MEFS WT as compared with SV40IM-MEFS KO Apaf1 changed from 81.32±1.06% to 72.23±3.08% at 5 µM oligomycin and from 17.72±0.62% to 5.96±0.2% at 50 µM oligomycin. The cell viability of SIM-MEFS WT Apaf1 as compared with SIM-MEFS KO Apaf1 changed from 84.13±1.89% to 72.23±2.52% at 5 µM oligomycin and from 43.01±2.67% to 17.89±6.86% at 50 µM oligomycin.

Next we performed a series of analyses for the characterisation of both SV40IM- and SIM-MEFS KO Apaf1. The proteins of the Bcl-2 family have been described to participate in MOMP regulation [21]. It has been reported that SV40IM-MEFS KO Apaf1 exhibits a lower expression of anti-apoptotic proteins Bcl-2 and Bcl-X_L_ [9], which might be related to the differences observed in Cyt *c* release. We found that not only SV40IM-MEFS KO Apaf1, but also SIM-MEFS KO Apaf1 show low levels of Bcl-2 and Bcl-X_L_ (Fig. 3A). In addition, SV40IM- and SIM-MEFS KO Apaf1 present a fragmented mitochondrial network (Fig. 3B), which correlates well with recently reported data and suggests that in SV40IM-MEFS KO Apaf1, the fission protein Drp1 localises in the mitochondria to a greater extent than in MEFS WT, thus favouring a fragmented mitochondrial network [9]. Next, we examined the ultra-structure of mitochondria by transmission electron microscopy (TEM). The mitochondria in the control MEFS WT cells were rounded with well visible cristae (Fig. 3C - black arrows in the WT-labelled panels). However, the mitochondria in the Apaf1-deficient cells (in both SV40IM- and SIM-MEFS KO Apaf1) were rather elongated and thinner (Fig. 3C - black arrows in the KO-labelled panels). Mitochondrial morphology changes have been linked to alterations of the cell's metabolic status [22]. These observations have been confirmed by the quantification of mitochondrial dimensions. Since samples were cut in the preparation process (see Methods), we assessed only mitochondrial width. In SV40IM-MEFS KO Apaf1, mitochondrial width decreased by an average 33% when compared to SV40IM-MEFS WT (Fig. 3C, right panel), while a 52% reduction in width was obtained in SIM-MEFS KO Apaf1 when compared to SIM-MEFS WT (Fig. 3C, right panel). It was also interesting to notice that the mitochondria in the MEFS WT control cells were mostly in the ‘orthodox’ state [23], whereas they were in a ‘condensed’ state, with greater electron density in the matrix, in MEFS KO Apaf1. These two different mitochondria ‘states’ have been previously characterised. In fact, ‘mitochondria states’ are related to mitochondrial status, and mitochondria populate one ‘state’ or the other depending on ADP availability. Mitochondria display ‘condensed’ conformation when ADP is in excess, but they revert to the ‘orthodox’ state when ADP is limiting [24], [25]. Then the TEM analysis suggests that in both SV40IM-MEFS KO Apaf1 and SIM-MEFS KO Apaf1, mitochondria are more active than in the control MEFS WT cells. In fact, we analysed the levels of intracellular ATP in SV40IM- and in SIM-MEFS KO Apaf1 to find that the two cell lines showed a significantly higher ATP content when compared to SV40IM- and SIM-MEFS WT (Fig. 4A). In SV40IM MEFS KO Apaf1, ATP almost doubled the content of the control cells (from 100.0±0.0 in SV40IM-MEFS WT to 196.6±28.42), while a 60% increase was observed in SIM-MEFS KO Apaf1 (from 100.0±0.0 in SIM MEFS WT to 161.8±3.740).

ATP production can vary to match energy demands [26]. ATP is produced from carbon fuels through glycolysis in the cytosol and via oxidative phosphorylation (OxPhos) in mitochondria. Glycolysis produces pyruvate for OxPhos under non-stressful conditions to generate 2 moles of ATP from 1 mole of glucose. However under stress due to a sudden drop in intracellular ATP, cells accelerate glycolysis through which the ATP generation rate becomes almost 100 times faster than that of OxPhos, be it with poor ATP production efficiency. Consequently, it leads to increased lactate production from pyruvate [27]. Then if the structural differences found in the mitochondria in MEFS KO Apaf1 can affect OxPhos, we should find increased lactate/pyruvate (L/P) ratios in these cells. In fact, it has been formerly reported that upon apoptotic treatment, the cell metabolism in the apoptosome-deficient cells is maintained by glycolysis [28]. We found that this was indeed the case in SV40IM-MEFS KO Apaf1 (Fig. 4B), suggesting that the main ATP source for these cells should be based on an increased glycolytic rate [29]. Conversely in the SIM-MEFS KO Apaf1 cells, we found that the L/P ratio was slightly lower as compared to the SIM-MEFS WT cells, suggesting the major relevance of OxPhos in ATP production in these cells. Next we reasoned as follows: if SIM-MEFS KO Apaf1 cells depend on OxPhos for ATP production, while SV40IM-MEFS KO Apaf1 cells rely on the glycolytic rate, then SIM-MEFS KO Apaf1 cells should be more sensitive to oligomycin (an inhibitor of mitochondrial ATP synthase) than the SV40IM-MEFS KO Apaf1 cells. Therefore, both KO Apaf1 cells were treated with oligomycin, while mitochondrial activity was evaluated by MTT. Dependence on ATP synthase can be clearly seen when comparing the results with the respective MEFS WT cells. The SV40-MEFS KO Apaf1 cells were more resistant to oligomycin than the control SV40-MEFS WT (Fig. 4C). However, SIM-MEFS KO Apaf1 cells were slightly more sensitive to the drug than the SIM-MEFS WT control cells (Fig. 4C).

In conclusion, we analysed mitochondrial features and cell death response to etoposide in two different Apaf1-deficient MEFS that differ in the immortalisation protocol. Unexpectedly, both, MEFS KO Apaf1 immortalised with the SV40 antigen (SV40IM-MEFS Apaf1) and those that spontaneously immortalised (SIM-MEFS Apaf1), presented relevant structural differences at the mitochondria when compared to MEFS WT, indicating a role for Apaf1 at the mitochondria. In fact when Apaf1 was absent, the mitochondria appeared more fragmented, elongated and thinner when compared to the MEFS WT mitochondria from animals with the same genetic background (SV40IM-MEFS WT and SIM-MEFS WT). These irregular mitochondria were predominantly found in a ‘condensed’ state and contained higher ATP levels than the counterpart mitochondria in MEFS WT. However, the origin of this ATP accumulation in the mitochondria was not clearly revealed, provided that the SV40IM-MEFS KO Apaf1 cells showed a more marked dependence on ATP production with high glycolytic rates, while SIM-MEFS KO Apaf1 were more dependent on OxPhos. Furthermore, etoposide-treated SV40IM-MEFS KO Apaf1 released more Cyt *c* and showed more cells with low ΔΨm than the counterpart cells from the WT animals. These cells died by caspase-independent processes, which were partially inhibited by Nec. In contrast, the SIM-MEFS KO Apaf1 cells were resistant to etoposide treatments as they maintained ΔΨm and showed minimal Cyt *c* release.

Hence the genetic background and immortalisation protocol might influence cells' response to death signals. Genetic ablation of Apaf1 dampens mitochondria which, depending on the genetic background, make cells more or less sensitive to DNA-inducing agents such as etoposide which, in sensitive cells, probably induces necroptosis when the canonical apoptosis components are not available. These studies, together with previous reports [6], [9], [11], illustrate not only the complex role that Apaf1 might play in cells, but also the importance of unravelling the network of signalling pathways that converges in the key proteins controlling apoptosis in probably not only cell death, but also in cell homeostasis. Through direct binding or accessory proteins, the results indicate a role for Apaf1 in the structural (and functional) arrangement of homeostatic mitochondria. It would be interesting to perform a thorough identification of the molecular mechanism as to how Apaf1 affects the mitochondria given their relevance and central position in cell fate decisions.

## Supporting Information

Figure S1 **ABT737 treatment induces cell death in SV40IM WT MEFS but not SV40IM KO MEFS and SIM MEFS.** (A) Percentage of cell survival measured by the trypan blue exclusion assay in SV40IM and SIM MEFS, WT and Apaf1 depleted, in the presence or absence of ABT737 (20 µM) for 24 h. (B) Caspase-3 like activity was measured under the same conditions described above. (C) Cells with Cyt c released measured by the flow cytometry analysis after incubation with ABT737 (20 µM) for 24 h. In all cases, bars represent the mean of three experiments ± s.d.
(TIF)
