# Supplementary figures and images for: Altered Mitochondria Morphology and Cell Metabolism in Apaf1-Deficient Cells
Source: PLoS One. 2014 Jan 9;9(1):e84666. doi: 10.1371/journal.pone.0084666 (PMC3886985; doi:10.1371/journal.pone.0084666)

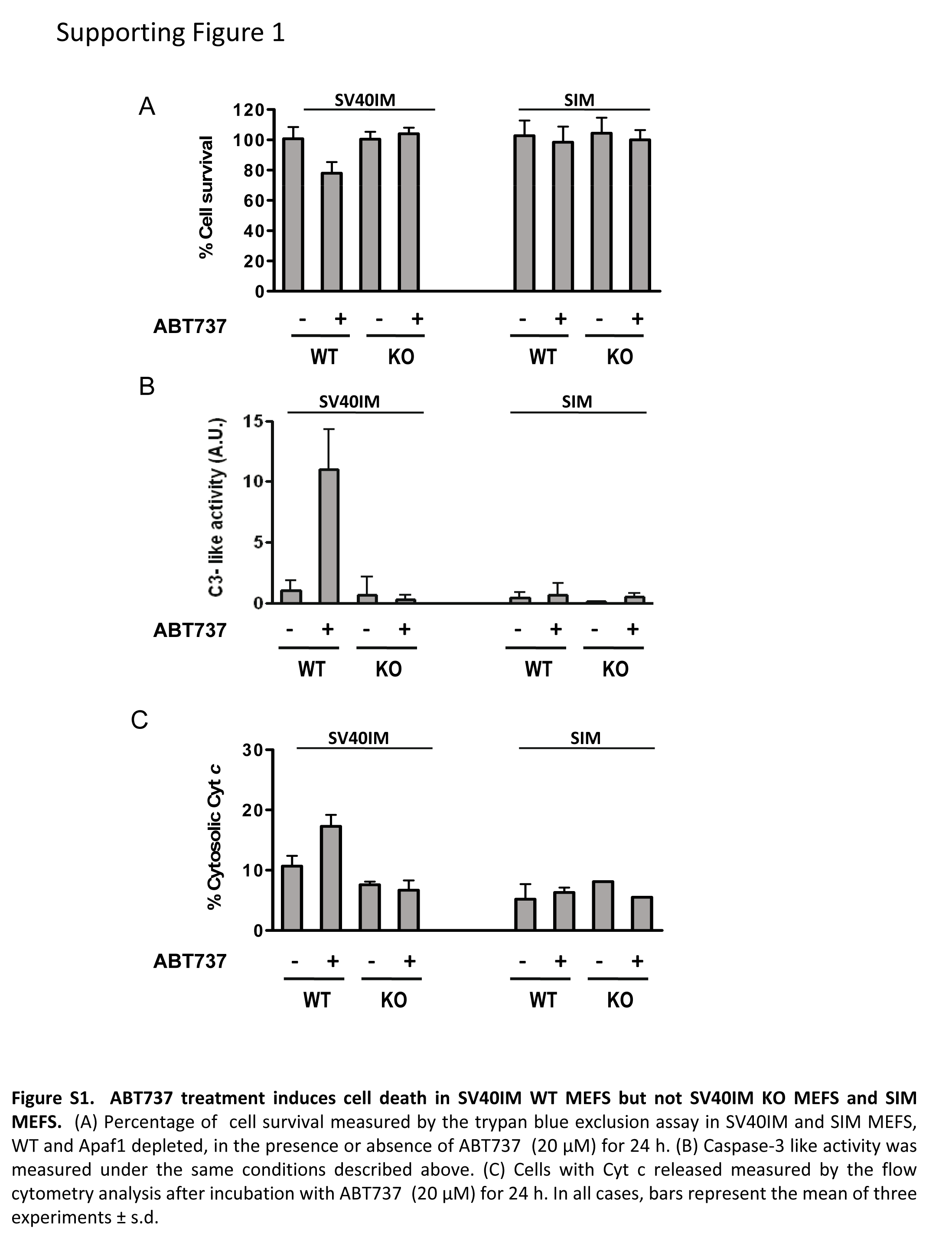

Supplement: Figure S1 — ABT737 treatment induces cell death in SV40IM WT MEFS but not SV40IM KO MEFS and SIM MEFS. (A) Percentage of cell survival measured by the trypan blue exclusion assay in SV40IM and SIM MEFS, WT and Apaf1 depleted, in the presence or absence of ABT737 (20 µM) for 24 h. (B) Caspase-3 like activity was measured under the same conditions described above. (C) Cells with Cyt c released measured by the flow cytometry analysis after incubation with ABT737 (20 µM) for 24 h. In all cases, bars represent the mean of three experiments ± s.d. (TIF) [file pone.0084666.s001.tif]
